# Urinary Fatty Acid Binding Protein 3 Has Prognostic Value in Peripheral Artery Disease

**DOI:** 10.3389/fcvm.2022.875244

**Published:** 2022-06-20

**Authors:** Ben Li, Abdelrahman Zamzam, Muzammil H. Syed, Niousha Jahanpour, Shubha Jain, Rawand Abdin, Mohammad Qadura

**Affiliations:** ^1^Division of Vascular Surgery, St. Michael's Hospital, Unity Health Toronto, University of Toronto, Toronto, ON, Canada; ^2^Department of Medicine, McMaster University, Hamilton, ON, Canada; ^3^Department of Surgery, University of Toronto, Toronto, ON, Canada; ^4^Keenan Research Center for Biomedical Science, Li Ka Shing Knowledge Institute, St. Michael's Hospital, Unity Health Toronto, University of Toronto, Toronto, ON, Canada

**Keywords:** fatty acid binding protein 3, urine, prognosis, peripheral artery disease, biomarker

## Abstract

**Background:**

Despite its significant association with limb loss and death, peripheral artery disease (PAD) remains underdiagnosed and undertreated. The current accepted gold-standard for PAD screening, the ankle brachial index (ABI), is limited by operator dependence, erroneous interpretation, and unreliability in patients with diabetes. Fatty acid binding protein 3 (FABP3) is an intracellular protein that becomes released into circulation and excreted into urine following skeletal muscle injury. We examined the prognostic ability of urinary FABP3 (uFABP3) in predicting adverse PAD-related events.

**Methods:**

In this prospective case-control study, urine samples were collected from patients with PAD (*n* = 142) and without PAD (*n* = 72). The cohort was followed for 2 years. uFABP3 was normalized to urinary creatinine (uCr) (uFABP3/uCr). The primary outcome was major adverse limb event (MALE; composite of vascular intervention [open or endovascular] or major limb amputation). The secondary outcome was worsening PAD status (drop in ABI≥0.15). Cox regression analyses with multivariable adjustment for baseline demographic and clinical variables were performed to assess the prognostic value of uFABP3/uCr with regards to predicting MALE and worsening PAD status.

**Results:**

Patients with PAD had significantly higher median [IQR] uFABP3/uCr levels (3.46 [2.45–6.90] vs. 2.61 [1.98–4.62], *p* = 0.001). MALE and worsening PAD status were observed in 21 (10%) and 28 (14%) patients, respectively. uFABP3/uCr predicted MALE and worsening PAD status with adjusted hazard ratios (HR) of 1.28 (1.16–1.41, *p* = 0.001) and 1.16 (1.02–1.27, *p* = 0.021), respectively. Patients with high uFABP3/uCr had a lower 2-year freedom from MALE (86 vs. 96%, *p* = 0.047) and worsening PAD status (78 vs. 99%, *p* = 0.001). There was good discriminatory ability for uFABP3/uCr in predicting the primary outcome of MALE, with an area under the receiver operating characteristics curve (AUROC) of 0.78.

**Conclusions:**

Measuring uFABP3/uCr levels in patients with PAD can help identify those at high risk of adverse PAD-related events. This study highlights the prognostic value of uFABP3 in risk-stratifying individuals for further diagnostic vascular evaluation or aggressive medical management.

## Introduction

Peripheral artery disease (PAD) involves atherosclerosis of the lower extremity arteries and affects over 200 million people worldwide (1). Despite its significant association with limb loss and death, PAD remains undertreated (2). As a result, patients with PAD usually have worse long-term prognosis compared to patients with coronary artery disease (3). One reason for this is the lack of a validated prognostic biomarker for PAD (4). The ankle brachial index (ABI) is currently the only validated screening tool for PAD; however, it is limited by operator dependence, erroneous interpretation, and unreliability in patients with diabetes due to calcified vessels (5, 6). Furthermore, ABI's are rarely performed in the primary care setting due to generalists' lack of comfort with performing and interpretating this test (7). A recent survey found that 79% of primary care providers did not perform ABI's routinely in their clinical practice, citing time constraints, unavailability of skilled personnel, and complexity of result interpretation as major barriers (8). Most clinicians viewed alternative forms of diagnosis, such as a blood or urine test, as preferable to ABI's and would enhance diagnostic simplicity and efficiency (8). Therefore, the identification and validation of a biomarker for PAD may improve cardiovascular risk stratification through early initiation of aggressive medical management, close ambulatory follow up, and arterial interventions.

In addition, ABI's are poor predictors of PAD-related complications (9). Pasqualini et al. (2012) showed that both low and high ABI were associated with cardiovascular mortality (10). Furthermore, Hatmi et al. (2014) demonstrated that a low ABI only had a sensitivity of 64% for predicting adverse cardiovascular events (11). Identification of better prognostic markers for PAD-related complications would improve risk-stratification and allow clinicians to identify patients who require further diagnostic evaluation, close follow-up, and aggressive medical/surgical therapy. For example, the Cardiovascular Outcomes for People Using Anticoagulation Strategies (COMPASS) trial demonstrated a significant reduction in major adverse cardiovascular events for patients with atherosclerotic vascular disease taking low-dose rivaroxaban (2.5 mg oral twice daily) in addition in ASA (12). Therefore, identifying high-risk PAD patients using a novel prognostic biomarker can allow clinicians to better select patients for intensive medical therapy including COMPASS trial low-dose rivaroxaban.

Fatty acid binding protein 3 (FABP3) is an intracellular protein that is normally absent from plasma but becomes released into circulation and excreted into urine following skeletal muscle injury, which may occur due to ischemia from PAD (13). Previous groups have demonstrated that FABP3 is associated with mitochondrial dysfunction, metabolic syndrome, and cardiovascular disease (14, 15). We previously demonstrated that FABP3 is associated with both the presence of PAD and severity of disease (16, 17). Given that urine samples can be obtained non-invasively and require less health care resources/personnel to collect and test compared to blood specimens, we assessed the diagnostic and prognostic value of urinary FABP3 (uFABP3) in PAD.

## Methods

### Ethics Approval

This study received approval from the research ethics board at Unity Health Toronto, University of Toronto, Canada. Informed consent was obtained from all participants, and all method were carried out in accordance with the World Medical Association Declaration of Helsinki (18).

### Patient Recruitment

A single-center prospective case-control study was conducted. Consecutive patients with and without PAD presenting to vascular surgery ambulatory clinics at St. Michael's Hospital, University of Toronto between April-December 2019 were recruited. PAD was defined as ABI < 0.9 or toe brachial index (TBI) < 0.67 and absent/diminished pedal pulses (19). Patients with chronic kidney disease (estimated glomerular filtration rate <60 mL/min/1.73 m^2^), acute ischemia or acute on chronic limb threatening ischemia, or acute coronary syndrome, congestive heart failure, uncontrolled arrhythmia, or elevated troponin within the past 12 months were excluded. Patients with recent cardiac disease were excluded to reduce the risk of confounding because FABP3 can be released secondary to myocardial ischemia and troponin elevation.

### Demographic and Clinical Characteristics

A complete medical history, physical exam, ABI values and symptomatic status relating to PAD were collected for each patient prior to recruitment. Baseline demographic and clinical characteristics recorded included age, sex, and history of hypertension (systolic blood pressure ≥ 130 mmHg, diastolic blood pressure ≥80 mmHg, or taking blood pressure lowering therapy), dyslipidemia (total cholesterol >5.2 mmol/L, triglyceride >1.7 mmol/L, or taking lipid lowering therapy), diabetes (hemoglobin A1c ≥ 6.5% or taking an antidiabetic medication), smoking (current or past), coronary artery disease, congestive heart failure, and ABI (16). Definitions for cardiovascular risk factors were based on American College of Cardiology guidelines (20, 21). Risk-reduction medications recorded included statins, angiotensin converting enzyme inhibitors (ACE-I) or angiotensin II receptor blockers (ARB), beta blockers, diuretics, acetylsalicylic acid (ASA), antiplatelets other than ASA, and low-dose rivaroxaban (2.5 mg oral twice daily).

### Urinary Sample Collection and FABP3 Multiplex Assay

Mid-stream urine samples were collected, aliquoted, and stored at −80°C prior to analysis. Urine samples were thawed on ice prior to analysis. To determine the concentration of FABP3 levels in the urine, samples were examined in duplicate using the MILLIPLEX MAP Human Cardiovascular Disease Magnetic Bead Panel 1 (EMD-Millipore; Billerica, MA) (22). To minimize any inter-assay variability, all analyses were carried out on the same day. Sample intra-assay and inter-assay coefficients of variability were <10%, which meets the threshold for statistical acceptability (23). Prior to any sample analysis, Fluidics Verification and Calibration bead kits (Luminex Corp) (24) were used to calibrate the MagPix analyzer (Luminex Corp; Austin, Texas) (25). At least 50 beads for uFABP3 were acquired using Luminex xPonent software and analyzed using Milliplex Analyst software (v5.1; EMD-Millipore) (26).

### Normalization of UFABP3 to Urinary Creatinine

Urine creatinine (uCr) levels were measured at the core laboratory at St. Michael's Hospital, University of Toronto using the Beckman Coulter AU680 laboratory analyzer (Beckman Coulter; Pasadena, California) (27). The uFABP3 concentration was normalized to uCr using single-spot urine samples (uFABP3/uCr: μg/g). This allowed for adjustment in urinary concentration errors and differences in hydration status.

### Follow-Up and Outcomes

Outpatient clinic visits were performed at 12-months as well as at the end of the study. During these follow-up visits, ABI was recorded in addition to PAD symptomatic status, PAD related interventions “revascularization or amputation,” and any changes made to concomitant treatment. Information regarding PAD-related hospitalizations and emergency room admissions was also collected. The primary outcome was 2-year major adverse limb events (MALE), defined as need for vascular intervention (open or endovascular lower extremity revascularization) or major amputation (any lower extremity amputation above the ankle). The individual components of MALE were also investigated. The secondary outcome was 2-year worsening PAD, defined as ABI drop ≥0.15, which has previously been demonstrated to be clinically relevant (28–30).

### Statistical Analysis

Baseline demographic and clinical characteristics were summarized as means and standard deviations (SDs) or numbers and proportions. Baseline differences between groups were calculated using independent *t*-test for continuous variables and chi-square test for categorical variables. uFABP3/uCr levels were not normally distributed, as determined by the Kolmogorov–Smirnov test, and were summarized as medians and interquartile ranges (IQRs). Event rates for MALE, vascular intervention, major amputation, and worsening PAD status (drop in ABI ≥ 0.15) at 2 years were reported for the overall cohort and compared between PAD and non-PAD patients using chi-square test. Hazard ratios (HRs) and 95% confidence intervals (95% CIs) for events per one unit increase in uFABP3/uCr were calculated using univariable and multivariable models, which were adjusted for age, sex, hypertension, dyslipidemia, diabetes, smoking, coronary artery disease, congestive heart failure, medications (statins, ACE-I/ARB, beta blockers, diuretics, ASA, antiplatelets other than ASA, and low-dose rivaroxaban), and serum creatinine. To find the relationship between uFAB*P3*/uCr and PAD severity based on ABI, we measured uFAB*P3*/uCr levels in subgroups of patients with no PAD (ABI ≥ 0.90), mild PAD (ABI 0.89–0.75), moderate PAD (ABI 0.74–0.50), and severe PAD (ABI < 0.50) as defined by the European Society for Vascular Medicine (ESVM) guidelines and compared the groups using independent *t-test* using the no PAD group as a reference *standard* (31). We also performed subgroup analysis assessing the impact of baseline ABI on outcomes. Using the whole cohort, receiver operator curve (ROC) analysis was conducted to identify a cut-off value for normalized uFABP3/uCr which could stratify our cohort into low and high uFABP3/uCr groups. The cut-off value was chosen based on the highest yielded Youden index. The overall event free survival rates of both groups were displayed using Kaplan-Meier curves, and differences between curves were compared with log-rank test. Significance was set at a two-tailed *p* < 0.05. All analyses were carried out using SPSS software version 23 (SPSS Inc., Chicago, Illinois, USA) (32).

## Results

### Characteristics of Patients With and Without PAD

Two-hundred and fourteen patients were included (142 with PAD and 72 without PAD). Mean age was 68 (SD 11) Years and 33% were female, 67% had hypertension, and 83% were current or past smokers, with no differences between groups. Patients with PAD were more likely to have dyslipidemia (85 vs. 58%, *p* = 0.001), diabetes (39 vs. 10%, *p* = 0.001), and coronary artery disease (CAD) (35 vs. 19%, *p* = 0.02). A greater proportion of patients with PAD were taking statins (87 vs. 62%, p = 0.001) and ACE-I/ARB (59 vs. 39%). Our results demonstrate that 62% of patients with pad received ASA; however, only 3% received low-dose rivaroxaban (2.5 mg oral twice daily) (Table 1). At baseline, our data demonstrates that patients with PAD had significantly higher median [IQR] UFABP3/uCr levels (3.46 [2.45–6.90] vs. 2.61 [1.98–4.62], *p* = 0.001).

**Table 1:** Demographic and clinical characteristics of patients with and without peripheral artery disease (PAD).

	**Overall**	**Non-PAD**	**PAD**	***P***
	**(*n* = 214)**	**(*n* = 72)**	**(*n* = 142)**	
**Mean (SD)** ^**‡**^				
Age, years	68 (11)	68 (12)	69 (10)	0.36
Ankle brachial index	0.78 (0.26)	1.08 (0.09)	0.62 (0.16)	**0.001**
**Demographics/comorbidities:** ***N*** **(%)** ^**¶**^				
Sex, male	144 (67)	49 (68)	95 (67)	0.87
Hypertension	143 (67)	42 (58)	101 (72)	0.05
Dyslipidemia	162 (76)	42 (58)	120 (85)	**0.001**
Diabetes	62 (29)	7 (10)	55 (39)	**0.001**
Smoking (current and past)	178 (83)	55 (76)	123 (87)	0.06
Coronary artery disease	63 (30)	14 (19)	49 (35)	**0.018**
Congestive heart failure	5 (2)	1 (1)	4 (3)	0.46
**Medications:** ***N*** **(%)** ^**¶**^				
Statins	164 (79)	44 (62)	120 (87)	**0.001**
ACE-I/ARB	109 (52)	28 (39)	81 (59)	**0.007**
Beta blockers	61 (29)	19 (27)	42 (31)	0.56
Diuretics	16 (8)	3 (4)	13 (10)	0.17
ASA	123 (58)	35 (49)	88 (62)	0.06
Antiplatelets (Other than ASA)	41 (19)	11 (15)	30 (21)	0.30
Rivaroxaban (low dose 2.5 mg oral twice daily)	4 (2)	0 (0)	4 (3)	0.15

‡ *Compared using independent t-test*;

¶ *Compared using chi-square test; ACE-I, angiotensin converting enzyme inhibitor; ARB, angiotensin II receptor blocker; ASA, acetylsalicylic acid. Bold values represent statistically significant values (p < 0.05)*.

### Two-Year Follow-Up and Clinical Event Rates

Complete 2-year follow-up was available for 201/214 (94%) patients, with a mean duration of 21.4 (SD 5.4) months. Overall, 21/214 (10%) patients had MALE (21/142 [15%, PAD] vs. 0 [non-PAD], *p* = 0.001) and 28/214 (14%) developed worsening PAD status (28/142 [22%, PAD] vs. 0 [non-PAD], *p* = 0.001). Furthermore, 18/214 (8%) required vascular intervention (18/142 [13%, PAD] vs. 0 [non-PAD], *p* = 0.002) and 3/214 (1%) underwent major amputation (3/142 [2%, PAD] vs. 0 [non-PAD], *p* = 0.21) (Table 2).

**Table 2:** Event rates for primary and secondary outcomes.

	**Overall**	**Non-PAD**	**PAD**	*P* ^*^
	**(*n* = 214)**	**(*n* = 72)**	**(*n* = 142)**	
	***N* (%)**	***N* (%)**	***N* (%)**	
MALE	21 (10)	0 (0)	21 (15)	**0.001**
Vascular intervention	18 (8)	0 (0)	18 (13)	**0.002**
Major amputation	3 (1)	0 (0)	3 (2)	0.21
Worsening PAD (drop in ABI ≥ 0.15)	28 (14)	0 (0)	28 (22)	**0.001**

*MALE, major adverse limb event: composite of vascular intervention and major amputation; PAD, peripheral artery disease; ABI, ankle brachial index*;

* *Compared using chi-square test. Bold values represent statistically significant values (p < 0.05)*.

### Cox Regression Analysis of Association Between uFABP3/uCr and PAD-Related Outcomes

There was a significant association between one unit increase of uFABP3/uCr and MALE (unadjusted HR 1.24 [95% CI 1.10–1.40], *p* = 0.001; adjusted HR 1.28 [95% CI 1.16–1.41], *p* = 0.001), need for vascular intervention (unadjusted HR 1.15 [95% CI 1.02–1.28], *p* = 0.031; adjusted HR 1.13 [95% CI 1.02–1.26], *p* = 0.034), and worsening PAD status (unadjusted HR 1.15 [95% CI 1.04–1.27], *p* = 0.009; adjusted HR 1.16 [95% CI 1.02–1.27], *p* = 0.021). There was no significant association between uFABP3/uCr and major amputation (unadjusted HR 1.19 [95% CI 0.92–1.55], *p* = 0.19; adjusted HR 1.19 [95% CI 0.81–1.76], *p* = 0.32) (Table 3).

**Table 3:** Hazard ratios for events per one unit increase in uFABP3/uCr.

	**Unadjusted**	***P***	**Adjusted**	***P***
	**HR (95% CI)**		**HR (95% CI)^‡^**	
MALE	1.24 (1.10–1.40)	**0.001**	1.28 (1.16–1.41)	**0.001**
Vascular intervention	1.15 (1.02–1.28)	**0.031**	1.13 (1.02–1.26)	**0.034**
Major amputation	1.19 (0.92–1.55)	0.19	1.19 (0.81–1.76)	0.32
Worsening PAD (drop in ABI ≥ 0.15)	1.15 (1.04–1.27)	**0.009**	1.16 (1.02–1.27)	**0.021**

*MALE, major adverse limb event: composite of vascular intervention and major amputation; PAD, peripheral artery disease; ABI, ankle brachial index*.

‡ *Adjusted for age, sex, hypertension, dyslipidemia, diabetes, smoking, coronary artery disease, congestive heart failure, medications (statins, ACE-I/ARB, beta blocker, diuretic, ASA, antiplatelets other than ASA, and low-dose rivaroxaban), and serum creatinine. Bold values represent statistically significant values (p < 0.05)*.

### Risk Stratification of Patients Based on uFABP3/uCr Levels

Based on receiver operating characteristic (ROC) curve analysis, our data demonstrates an ideal uFABP3/uCr cut-off value for PAD of 2.70 using a Youden index of 54%. We observed a good discriminatory ability of uFABP3/uCr for predicting the primary outcome of MALE with an area under the curve (AUC) of 0.783, sensitivity of 86%, and specificity of 68%. To investigate the potential of uFABP3/uCr in risk stratifying patients, the uFABP3/uCr cut-off value based on ROC curve analysis was used to stratify patients into two groups: low uFABP3/uCr (≤ 2.70 μg/g; *n* = 79) and high uFABP3/uCr (>2.70 μg/g; *n* = 135). Patients with high uFABP3/uCr were older (70 [SD 9] vs. 65 [SD 12] years, *p* = 0.003), had a lower mean ABI (0.73 [SD 0.24] vs. 0.84 [SD 0.26], *p* = 0.006), and were more likely to be diagnosed with dyslipidemia (81 vs. 68%, *p* = 0.035) and PAD (83 vs. 38%, *p* = 0.001). There were no differences in risk reduction medications between groups, with only 2% of patients with high uFABP3/uCr taking low-dose rivaroxaban.

Over the study 2-year period, patients with high uFABP3/uCr were more likely to develop MALE (14 vs. 4%, *p* = 0.034), require a vascular intervention (12 vs. 4%, *p* = 0.046), and have worsening PAD (22 vs. 1%, *p* = 0.001). There was no difference in major amputation rates between groups (2 vs. 0%, *p* = 0.18) (Table 4).

**Table 4:** Demographic and clinical characteristics of patients with low and high uFABP3/uCr.

	**Low uFABP3/uCr**	**High uFABP3/uCr**	***P***
	**(*n* = 79)**	**(*n* = 135)**	
**Mean (SD)** ^**‡**^			
Age, years	65 (12)	70 (9)	**0.003**
Ankle brachial index	0.84 (0.26)	0.73 (0.24)	**0.006**
**Demographics/comorbidities:** ***N*** **(%)** ^**¶**^			
Peripheral artery disease	35 (38)	112 (83)	**0.001**
Sex, male	58 (73)	86 (64)	0.18
Hypertension	46 (59)	97 (72)	0.06
Dyslipidemia	53 (68)	109 (81)	**0.035**
Diabetes	17 (22)	45 (33)	0.07
Smoking (current and past)	66 (84)	112 (83)	0.91
Coronary artery disease	22 (28)	41 (31)	0.67
**Medications:** ***N*** **(%)** ^**¶**^			
Statins	56 (74)	108 (81)	0.20
ACE-I/ARB	34 (45)	75 (57)	0.09
Beta blockers	21 (28)	40 (30)	0.68
Diuretics	5 (7)	11 (8)	0.64
ASA	44 (56)	79 (59)	0.69
Antiplatelets (Other than ASA)	17 (22)	24 (18)	0.50
Rivaroxaban (low dose 2.5 mg oral twice daily)	1 (1)	3 (2)	0.62
**Events:** ***N*** **(%)** ^**¶**^			
MALE	3 (4)	19 (14)	**0.034**
Vascular intervention	3 (4)	16 (12)	**0.046**
Major amputation	0 (0)	3 (2)	0.18
Worsening PAD (drop in ABI ≥ 0.15)	1 (1)	27 (22)	**0.001**

*ACE-I, angiotensin converting enzyme inhibitor; ARB, angiotensin II receptor blocker; ASA, acetylsalicylic acid; MALE, major adverse limb event: composite of vascular intervention and major amputation; PAD, peripheral artery disease; ABI, ankle brachial index.; uFABP3/uCr [urinary fatty acid binding protein 3 (uFABP3) normalized to urinary creatinine (uCr)]; Low uFABP3/uCr defined as ≤ 2.70 μg/g; High uFABP3/uCr defined as >2.70 μg/g*;

‡ *Compared using independent t-test*;

¶ *Compared using chi-square test. Bold values represent statistically significant values (p < 0.05)*.

### Two-Year Event-Free Analysis in Patients With Low and High uFABP3/uCr

**Figure 1:**
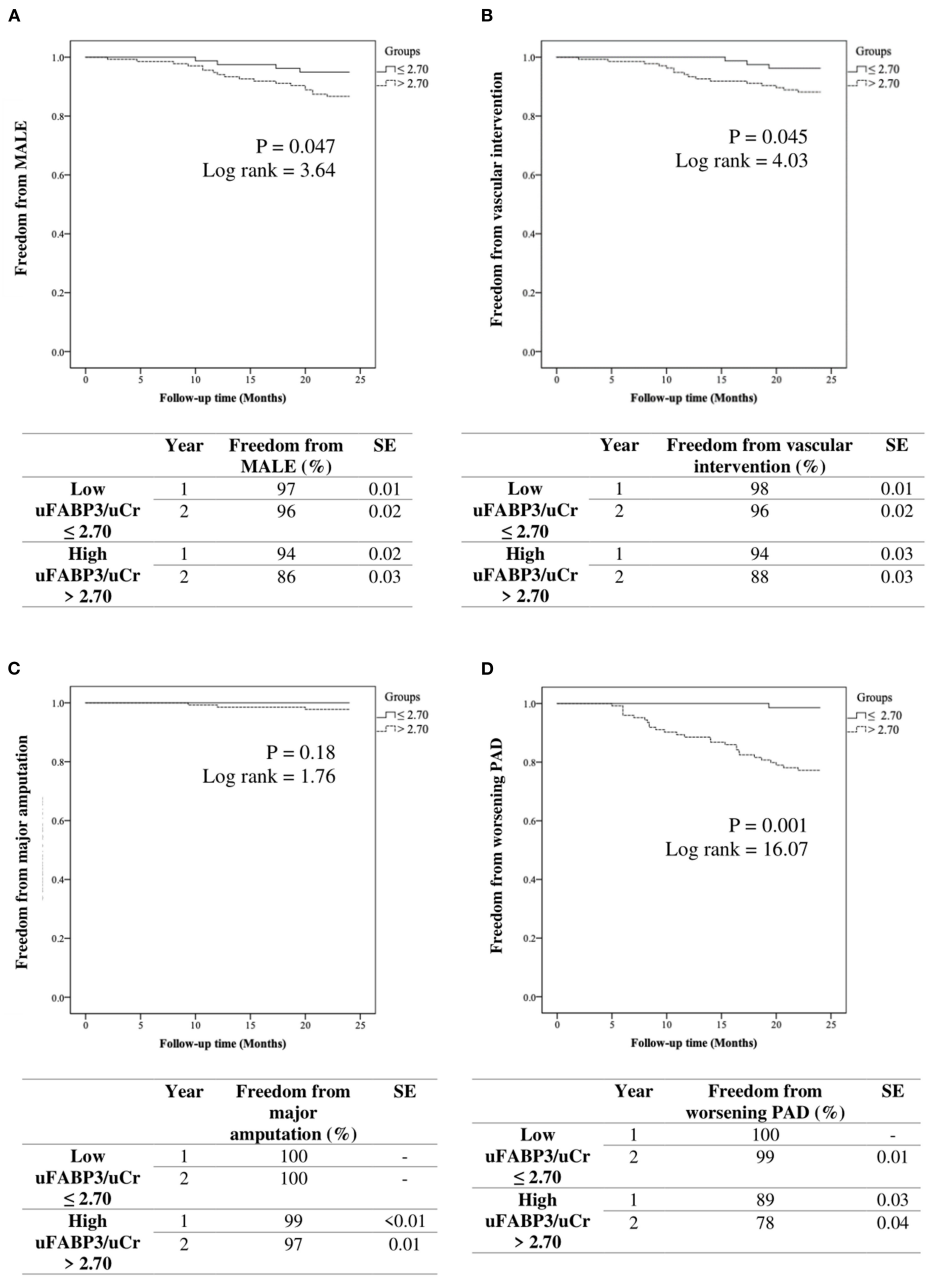
Kaplan-Meier analysis of event free survival rates in patients with low vs. high urinary fatty acid binding protein 3 normalized to urinary creatinine (uFABP3/uCr) for **(A)** MALE (major adverse limb event), **(B)** vascular intervention, **(C)** major amputation, and **(D)** worsening PAD (ankle brachial index drop ≥ 0.15). SE (standard error).

MALE-free survival rates at 1 year and 2 years were 97, and 96% in the low uFABP3/uCr group and 94, and 86% in the high uFABP3/uCr group (*p* = 0.047; log rank = 3.64). Freedom from vascular intervention at 1 year and 2 years were 98, and 96% in the low uFABP3/uCr group and 94, and 88% in the high uFABP3/uCr group (*p* = 0.045; log rank = 4.03). On the other hand, there was no significant difference in the event-free survival rate for major amputations between low and high uFABP3/uCr groups (*p* = 0.18; log rank = 1.76). Event-free survival rates for worsening PAD status at 1 year and 2 years were 100% and 99% in the low uFABP3/uCr group and 89, and 78% in the high uFABP3/uCr group (*p* = 0.001; log rank = 16.07) (Figure 1).

### Subgroup Analysis of uFABP3/uCr Levels and Outcomes Based on PAD Severity

There was a significant correlation between median uFABP3/uCr levels and PAD severity: (ABI ≥ 0.90 [2.61 (IQR 1.98–4.62)], ABI 0.89 – 0.75 [3.13 (IQR 2.61 – 5.49), p = 0.047], ABI 0.74–0.50 [3.55 (IQR 2.42–6.94), *p* = 0.002], ABI < 0.50 [3.99 (IQR 2.32–7.12), p = 0.002]). Patients with no PAD did not develop any primary or secondary outcomes. Compared to patients with mild PAD, those with moderate PAD were more likely to develop MALE (62 vs. 29%), require vascular intervention (50 vs. 39%), and have worsening PAD status (54 vs. 46%). Since most patients with acute on chronic ischemia who typically have an ABI < 0.5 were excluded from the study, we recruited few patients with severe PAD. In this subset of patients, we noted a small proportion of individuals with severe PAD (ABI < 0.5) who suffered adverse events [MALE (10%), vascular intervention (11%), major amputation (33%), and worsening PAD status (0%)] (Supplementary Table 1).

## Discussion

Our single-center prospective case-control study demonstrates that uFABP3/uCr has diagnostic and prognostic value in PAD. We identified a cohort of 214 patients with and without PAD and demonstrated that uFABP3/uCr was elevated in the PAD group. Furthermore, higher levels of uFABP3/uCr predicted future PAD-related complications. Interestingly, most of the observed adverse events occurred in patients with moderate PAD based on ABI rather than patients with severe PAD, therefore our data suggests that ABI at baseline is not a good predictor of future adverse events, as corroborated by previous work by other groups (10, 11). This prompted us to risk stratified our cohort into low and high uFABP3/uCr based on ROC analysis. Our data demonstrated that patients with high uFABP3/uCr had a lower mean ABI and were more likely to be diagnosed with PAD. Finally, we showed that patients with high uFABP3/uCr were more likely to progress in their PAD disease state and require vascular intervention over a 2-year period. Overall, uFABP3/uCr had good discriminatory ability to predict our primary outcome of MALE, with an AUROC of 0.78. This is the first study validating a urinary biomarker for the prognosis of PAD in humans.

FABP3 belongs to a family of multi-gene fatty acid binding proteins (FABP's) and is primarily expressed in skeletal muscle and myocardial tissue (33). FABP3 is found predominantly intracellularly within the cytosol, where it mediates the uptake and transport of fatty acids (34). Previous studies have demonstrated the release of FABP3 from skeletal muscle in patients following exercise or muscle injury (13, 35, 36). In a similar context, patients with PAD experience muscle ischemia either transiently (claudication) or constantly (chronic limb threatening ischemia) (37, 38). This leads to FABP3 release into circulation and eventual excretion into the urine (39, 40). Others have demonstrated that FABP3 is associated with endothelial dysfunction, which may result from PAD (41). These are potential biochemical pathways by which FABP3 acts as a biomarker for PAD.

Previous studies have examined FABP's as biomarkers for other disease states. Ozawa et al. showed that FABP3 may be a potential mediator for diabetic nephropathy in murine models (42). Hayashida et al. demonstrated that serum and urine FABP3 may be early markers of myocardial injury in patients undergoing cardiac surgery (43). Others have investigated urinary FABP's as biomarkers for patients with acute kidney injury requiring renal replacement therapy (44). Given accumulating evidence demonstrating the value of FABP's in acting as biomarkers for life-threatening diseases, ongoing study in this area is warranted, particularly with a view to translate research findings into clinical practice to improve patient outcomes through early diagnosis and treatment. This is the first study to demonstrate the prognostic value of uFABP3/uCr in PAD patients.

There are several potential explanations for our findings. First, we demonstrated that PAD patients are more likely to have coronary artery disease (CAD) due to a greater burden of related cardiovascular risk factors including dyslipidemia and diabetes. This corroborates previous evidence demonstrating that individuals with PAD often have widespread arterial disease, with 61% of PAD patients being diagnosed with co-morbid CAD (45). As a result, they are at a 6-fold increased risk of cardiovascular death compared to patients without PAD (46). This highlights the importance of developing a widely applicable screening and prognostic tool for PAD. Second, we found that uFABP3/uCr levels were higher in PAD patients. This is likely because muscle ischemia secondary to PAD leads to release of uFABP3 into circulation, which then becomes excreted into urine. This provides a valuable biomarker that can be measured non-invasively in patients to screen for PAD. Third, patients with high uFABP3/uCr were more likely to develop MALE, require vascular intervention, and have worsening PAD over a 2-year period. There was also an association between every one unit increase in uFABP3/uCr and PAD-related complications. This suggests that uFABP3/uCr not only has diagnostic value, but also provides prognostic information regarding disease trajectory. Importantly, we demonstrated that high uFABP3/uCr was associated with worse PAD prognosis independent of CAD. Specifically, patients with active CAD were excluded from our analysis (acute coronary syndrome, congestive heart failure, uncontrolled arrhythmia, or elevated troponin within the past 12 months), which significantly reduced the risk of confounding. Even after adjusting for comorbidities including CAD and diabetes, there remained an independent association between uFABP3/uCr and MALE / worsening PAD status, which corroborates previous findings from our group (17). Therefore, this biomarker has the potential to risk stratify patients and guide healthcare providers with regards to aggressiveness of medical and surgical therapy, intensity of follow-up, and allocation of health care resources. Less than 5% of patients with PAD in our study were taking low-dose rivaroxaban, which has been demonstrated to provide significant cardiovascular benefit in patients with atherosclerotic vascular disease based on the COMPASS trial (12). Furthermore, only 62% and 87% of patients with PAD were taking ASA and statins respectively, despite these risk-reduction medications being Grade 1A recommendations for cardiovascular mortality and limb salvage benefit based on American Heart Association guidelines (47). Therefore, there are significant gaps in medical management of PAD, which may be addressed through a prognostic biomarker that can improve identification of high-risk patients that require more intensive therapy. Fourth, there was likely no difference in 2-year major amputation between groups due to the low incidence of this event (*n* = 3/214) as well as the fact that patients were followed closely in this prospective study and underwent early revascularization to prevent limb loss. Fifth, our subgroup analysis demonstrated a significant association between uFABP3/uCr and PAD severity based on ABI, which corroborates previous evidence from our group (17). Our analysis of outcomes based on PAD severity showed that patients with moderate PAD were more likely to develop adverse events compared to those with mild PAD or no PAD. Only a small number of patients with severe PAD (ABI < 0.5) were included in this study. This might be due to the fact that patients with acute on chronic limb ischemia who typically have an ABI < 0.5 were excluded from this study. These patients were excluded since they already possess some of the recorded events for the study, such as need for vascular intervention or limb amputation. Within this small subset of patients and relative to patients with moderate PAD, we noted that a small number of patients with severe PAD based on ABI developed MALE or worsening PAD. This information reflects the poor prognostic ability of baseline ABI in predicting future adverse events, as demonstrated by other groups (10, 11). However, larger clinical trials are needed to investigate the prognostic ability of ABI. Our study suggests that uFAB *P3*/uCr may be a better prognostic marker for adverse PAD-related events.

This study has several limitations. First, this was not a randomized study and there were differences in baseline characteristics between groups. However, our analysis adjusted for important baseline characteristics including age, sex, hypertension, dyslipidemia, diabetes, smoking, coronary artery disease, congestive heart failure, medications (statins, ACE-I/ARB, beta blocker, diuretic, ASA, antiplatelets other than ASA, and low-dose rivaroxaban), and serum creatinine. Second, 2-year outcomes are reported, and longer follow-up is needed to better understand the prognostic value of uFABP3/uCr given that PAD is generally a long-standing disease. Third, we excluded patients with eGFR <60, acute limb ischemia, or acute coronary syndrome, congestive heart failure, uncontrolled arrhythmia, or elevated troponin within the past 12 months. Therefore, the results may not apply to all patients with suspected PAD and further investigation of uFABP3 is needed to better understand how the biomarker can be effectively implemented as a screening and prognostic tool in clinical practice. Fourth, additional variables including body weight, lean body mass, percent body fat, lipid profiles, and echocardiogram data were not collected and may provide additional information regarding our patient population. The demographic and clinical variables reported in our study reflect clinically relevant indicators of the health of vascular surgery patients. Future study could collect more granular data to better understand the impact of baseline factors on PAD progression. Fifth, the major amputation rate was low in our study (*n* = 3/214) and likely contributed to statistically insignificant findings for this *second* ary outcome. We hypothesize that with longer follow-up and more events, our study would have detected a significant difference in major amputation rates based on uFAB *P3*/uCr levels.

## Conclusions

Our study demonstrates three important findings. First, elevated uFABP3/uCr is associated with PAD. Second, patients with high uFABP3/uCr are more likely to develop MALE, require vascular intervention, and have worsening PAD even after adjusting for CAD history. Finally, uFABP3/uCr predicts MALE with an AUROC of 0.78. These results suggest that uFABP3/uCr has good diagnostic and prognostic value in PAD independent of CAD. The severity of PAD both at baseline and over the study period were captured in our study. Measurement of uFABP3/uCr can improve risk-stratification and identify patients at high risk for developing PAD-related complications who require additional diagnostic evaluation, close follow-up, and aggressive medical management, including prescription of low-dose rivaroxaban. Given that urine samples are easily collected and analyzed in the primary care setting, this biomarker has potential for widespread clinical implementation as a PAD screening and prognostic tool. Larger studies, particularly clinical trials, should be performed to confirm our findings.

## Data Availability Statement

The original contributions presented in the study are included in the article/Supplementary Material, further inquiries can be directed to the corresponding author/s.

## Ethics Statement

The studies involving human participants were reviewed and approved by Unity Health Toronto Research Ethics Board, University of Toronto. The patients/participants provided their written informed consent to participate in this study.

## Author Contributions

SJ, NJ, and MS: acquisition, analysis, and interpretation of data, revising the manuscript for important intellectual content, and approval of the final manuscript draft submitted for publication. BL and AZ: methodology, statistical analysis, data analysis and interpretation, writing—original draft, revising the manuscript for important intellectual content, and approval of the final manuscript draft submitted for publication. RA and MQ: study concept and design, methodology, data analysis and interpretation, writing—original draft, revising the manuscript for important intellectual content, and approval of the final manuscript draft submitted for publication. All authors contributed to the article and approved the submitted version.

## Conflict of Interest

The authors declare that the research was conducted in the absence of any commercial or financial relationships that could be construed as a potential conflict of interest.

## Publisher's Note

All claims expressed in this article are solely those of the authors and do not necessarily represent those of their affiliated organizations, or those of the publisher, the editors and the reviewers. Any product that may be evaluated in this article, or claim that may be made by its manufacturer, is not guaranteed or endorsed by the publisher.
