# Supplementary material for: Urinary Fatty Acid Binding Protein 3 Has Prognostic Value in Peripheral Artery Disease
Source: Front Cardiovasc Med. 2022 Jun 20;9:875244. doi: 10.3389/fcvm.2022.875244 (PMC9250966; doi:10.3389/fcvm.2022.875244)
Supplement: Supplementary file 1 [file Table_1.pdf]

Supplemental Table 1: Subgroup analysis of event rates based on PAD severity

**ABI**	**≥ 0.90 [no PAD] (n=72)**	**0.89 – 0.75 [mild PAD] (n=36)**	**0.74 – 0.50 [moderate PAD] (n=76)**	**< 0.50 [severe PAD] (n=30)**
**MALE (n=21)**	0	6 (29)	13 (62)	2 (10)
**Vascular intervention (n=18)**	0	7 (39)	9 (50)	2 (11)
**Major amputation (n=3)**	0	1 (33)	1 (33)	1 (33)
**Worsening PAD status (n=28)**	0	13 (46)	15 (54)	0

*Results reported as N (%)
Abbreviations: ABI (ankle brachial index), PAD (peripheral artery disease), MALE (major adverse limb event)
